# Keeping the balance: Trade-offs between human brain evolution, autism, and schizophrenia

**DOI:** 10.3389/fgene.2022.1009390

**Published:** 2022-11-21

**Authors:** Eryk Duński, Aleksandra Pękowska

**Affiliations:** Dioscuri Centre for Chromatin Biology and Epigenomics, Nencki Institute of Experimental Biology, Polish Academy of Sciences, Warsaw, Poland

**Keywords:** schizophrenia, ASD, cognition, evolution, psychiatric discorders

## Abstract

The unique qualities of the human brain are a product of a complex evolutionary process. Evolution, famously described by François Jacob as a “tinkerer,” builds upon existing genetic elements by modifying and repurposing them for new functions. Genetic changes in DNA may lead to the emergence of new genes or cause altered gene expression patterns. Both gene and regulatory element mutations may lead to new functions. Yet, this process may lead to side-effects. An evolutionary trade-off occurs when an otherwise beneficial change, which is important for evolutionary success and is under strong positive selection, concurrently results in a detrimental change in another trait. Pleiotropy occurs when a gene affects multiple traits. Antagonistic pleiotropy is a phenomenon whereby a genetic variant leads to an increase in fitness at one life-stage or in a specific environment, but simultaneously decreases fitness in another respect. Therefore, it is conceivable that the molecular underpinnings of evolution of highly complex traits, including brain size or cognitive ability, under certain conditions could result in deleterious effects, which would increase the susceptibility to psychiatric or neurodevelopmental diseases. Here, we discuss possible trade-offs and antagonistic pleiotropies between evolutionary change in a gene sequence, dosage or activity and the susceptibility of individuals to autism spectrum disorders and schizophrenia. We present current knowledge about genes and alterations in gene regulatory landscapes, which have likely played a role in establishing human-specific traits and have been implicated in those diseases.

## 1 Introduction

Consciousness, self-awareness, and abstract thinking are the essential features of the human mind. One of the most fascinating questions in neurobiology is what changes in the genome have led to the emergence of unique properties of the human brain. However, the same shifts that have led to the development of the unprecedented capacities of the human brain may also underlie its susceptibility to disease. Neuropsychiatric disorders (NDs), in particular schizophrenia (SCZ) or autism spectrum disorder (ASD), specifically affect self-awareness, cognition, and the ability for social interaction (Geschwind and Rakic, 2013; Lai et al., 2014; Crespi and Ph, 2016; Owen et al., 2016; Benítez-Burraco et al., 2022), which were under positive pressure in human lineage (Kaczanowska et al., 2022). Remarkably, numerous genes that might underlie the unique features of the human brain are often at the same time risk genes of ASD or SCZ (see below). Loci associated with NDs are frequently expressed early in the brain development (O’Brien et al., 2018; Polioudakis et al., 2019; Ball et al., 2020; Satterstrom et al., 2020), the timepoint when the largest divergence of transcriptomic profiles between humans and macaques is also observed (Zhu et al., 2018). Together, these observations point to a possible link between evolutionary changes in brain biology and the emergence of SCZ and ASD.

ASD encompasses a broad group of conditions characterized by defective social communication, repetitive behaviour, and restricted interests or activities (Grzadzinski et al., 2013). Psychosis, which consists of altered perception of what is real and what is not, constitutes the primary feature of SCZ (Owen et al., 2016). It can be accompanied by social cognitive impairment and neurocognitive disfunctions (Stratton et al., 2017; Engelstad et al., 2019). Both SCZ and ASD affect the essential features of the human mind – intelligence and self-awareness (Keefe et al., 2005; Lord et al., 2018). Both diseases feature a strong genetic component. Heritability of SCZ is estimated between 47.3% and 81% (Owen et al., 2016; Chou et al., 2017) while for the ASD it is estimated between 64% and 91% (Tick et al., 2016). Despite a detrimental impact on reproductive fitness (Van Dongen and Boomsma, 2013) both SCZ and ASD are highly frequent in the human population and affect almost 1% (SCZ) (McGrath et al., 2008), and 1%–2% (ASD) of adults worldwide (Baio, 2012; Lai et al., 2014). These observations suggest that genes and gene variants linked to SCZ and ASD may play crucial roles in human brain biology; benefits from their functions may outweigh the negative selective pressure and the loss of fitness due to disease risk. Indeed, while ASD patients frequently display below-average intelligence quotient, they also feature enhanced perception, visuospatial performance, increased attention to detail and more focused concentration (Belmonte et al., 2004; Mottron et al., 2006; Kelleher and Bear, 2008; Muth et al., 2014; Sacco et al., 2015). Altogether, these observations led to a proposal that ASD may in fact, be considered a “disorder of high intelligence” (Crespi, 2016), which shares genetic basis with high cognitive abilities (Hagenaars et al., 2016). Furthermore, there is increasing evidence of overlapping genetic bases of ASD and SCZ (Fromer et al., 2014; Satterstrom et al., 2020), and that some aspects of ASD and SCZ may represent opposite ends of the same gene dosage spectrum. Therefore, it is interesting to consider the common genetic bases of evolution and susceptibility to SCZ and ASD (Crespi and Ph, 2016). Multiple microdeletion syndromes which are associated with both SCZ and ASD (Crespi et al., 2010) contain genes which have undergone an evolutionary change in humans (1q21.1 – *NOTCH2NL*, *NBPF* genes (Fiddes et al., 2018, 2019); 15q13.3–*GOLGA8* (Hahn et al., 2007; Bekpen and Tautz, 2019), 16p11.2–*BOLA2* (Zufferey et al., 2012; Nuttle et al., 2016)*;* 16p13.1–*NDE1* (Mosca et al., 2017; Monda and Cheeseman, 2018)) (Table 1). Taken together, the evidence points to the possibility that mechanisms which rendered human brain unique during evolution entail a trade-off of increased neuropsychiatric disease risk. In this mini-review article, we focus on the genetic link between evolution of the human brain and SCZ and ASD.

**TABLE 1:** Summary of genes which are implicated in ASD and SCZ, and bear signs of recent evolution in humans. Observed or proposed evolutionary trade-offs and antagonistic pleiotropies caused by evolution of those genes are listed.

Gene	Suggested affected trait in humans	Evolutionary change	Trade-off/pleiotropic effect	References
*NOTCH2NL (NOTCH2NLA, NOTCH2NLB, NOTCH2NLC, NOTCH2NLR)* and *NBPF* gene family (1–23)	Intellectual ability and brain size, by impacting the self renewal and clonal expansion capacity of neural stem cells	Gene family expansion (*NOTCH2NL* and *NBPF*) increase in the number of Olduvai domains in the genome (*NBPF*) reactivation of a gene (*NOTCH2NL*)	SCZ risk; ASDs risk; structural instability of chromosomal location	Brunetti-Pierri et al. (2008); Stone et al. (2008); Mefford et al. (2008); Searles Quick et al. (2015); Davis et al. (2014, 2015, 2019); Bernier et al. (2016); Zhang et al. (2016); Macé et al. (2017); Suzuki et al. (2018); Fiddes et al. (2018, 2019); Florio et al. (2018); Kanton et al. (2019); Pang et al. (2020)
*BOLA2* (16p11.2 locus)	Iron sulphur homeostasis, synaptic marker expression, neurophysiological properties of neurons	Family expansion	ASD risk, SCZ risk, microcephaly, cognitive impairment	Li and Outten, (2012); Zufferey et al. (2012); Banci et al. (2015); Nuttle et al. (2016); Wallace et al. (2018); Giannuzzi et al. (2019); Rein and Yan, (2020); Chung et al. (2021); Sundberg et al. (2021)
*NRG3*	Neurite outgrowth, glutamate release, ERBB4 regulation	Positive selection compared to Neanderthals	SCZ risk, ASD risk, severity of SCZ symptoms	Green et al. (2010); Zhang et al. (2016); Hayes et al. (2016); Mostaid et al. (2016); Paterson et al. (2017); Vullhorst et al. (2017); Bartolini et al. (2017); Avramopoulos, (2018); Müller et al. (2018); Wang et al. (2018); Rahman-Enyart et al. (2020); Zhou et al. (2020); Exposito-Alonso et al. (2020); Fairley et al. (2020); Li et al. (2020); Karlsson et al. (2021); Ahmad et al. (2022)
*CADPS2*	Regulation of social behavior, secretion of synaptic vesicles, secretion of BDNF and NT-3	Positive selection compared to Neanderthals	SCZ risk, ASD risk	International Molecular Genetic Study of Autism Consortium, (2001); Sadakata et al. (2004, 2007); Hattori et al. (2011); Shinoda et al. (2011); Bonora et al. (2014); Zhang et al. (2016); Grabowski et al. (2017); Shinoda et al., 2018, 2019; Fujima et al. (2021); Nagy et al. (2021)
*AUTS2*	Regulation of neuronal differentiation, cytoskeleton, regulation of excitatory synapse activation	Positive selection compared to Neanderthals	ASD risk	Sultana et al. (2002); Pollard et al. (2006a, 2006b); Prabhakar et al. (2006); Kalscheuer et al. (2007); Bakkaloglu et al. (2008); Bedogni et al. (2010); Pinto et al. (2010); Green et al. (2010); Huang et al. (2010); Schumann et al. (2011); Girirajan et al. (2011); Nagamani et al. (2013); Gao et al. (2014); Hori et al. (2014); Zhang et al. (2016); Karlsson et al. (2021); Monderer-Rothkoff et al. (2021); Castanza et al. (2021); Biel et al. (2022)
*AMBRA1*	Regulation of neurogenesis and autophagy	Positive selection compared to Neanderthals	ASD risk, SCZ risk	Maria Fimia et al. (2007); Rietschel et al. (2012); Heinrich et al. (2013); Prüfer et al. (2014); Yazdankhah et al. (2014); Cianfanelli et al. (2015); Mitjans et al. (2017); Nobili et al. (2018); La Barbera et al. (2019); Trajkova et al. (2020)

## 2 Brain evolution: Size and connectivity

**FIGURE 1:**
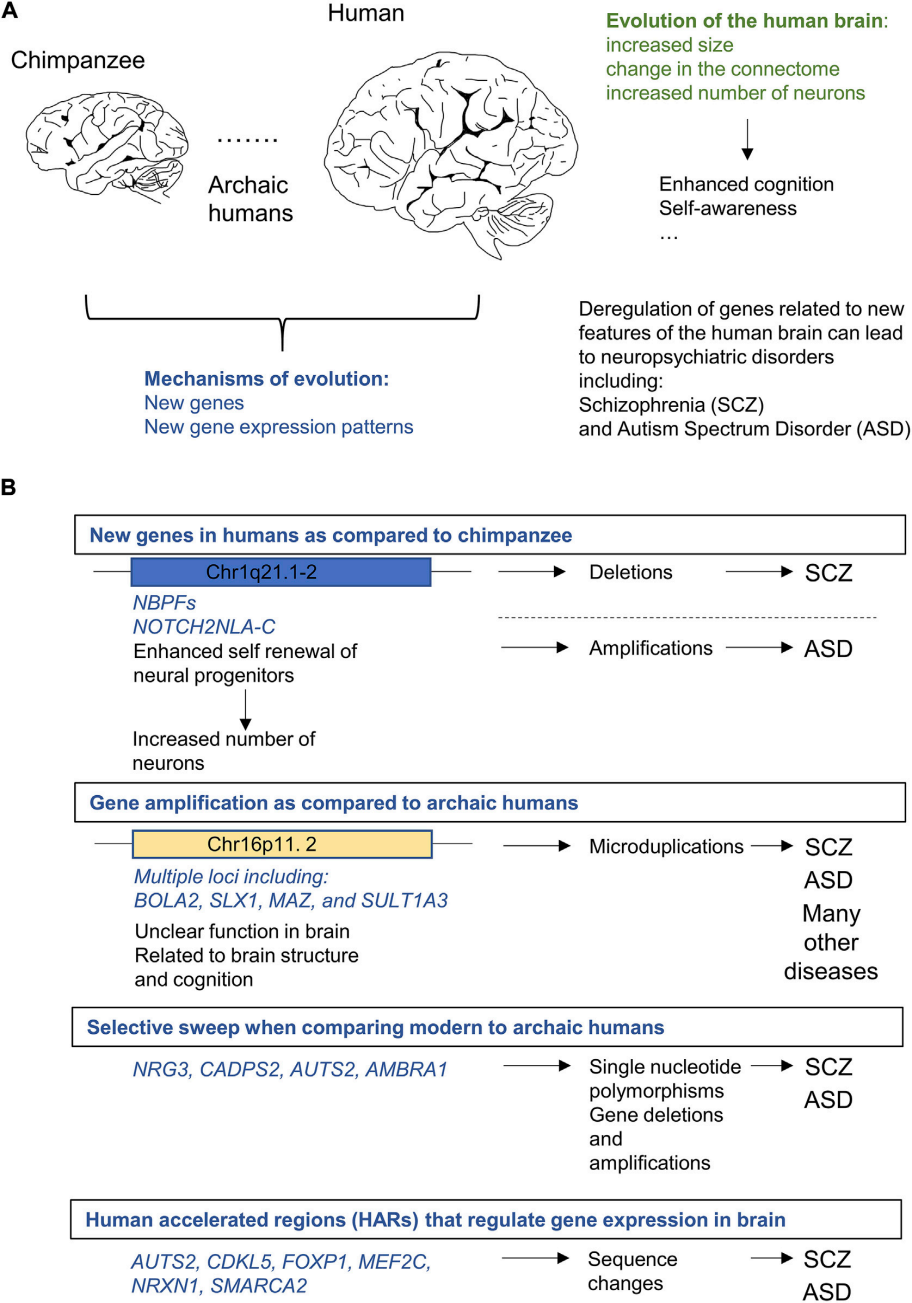
Genetic alterations in loci related to human brain evolution can lead to neuropsychiatric disorders specifically affecting self-awareness and cognition including schizophrenia (SCZ) and autism spectrum disorder (ASD). **(A)**. Human brain is larger and contains more neurons than the brain of our closest living relative the chimpanzee (*Pan troglodytes*). Evolution led to the enhancement of several features of human brain including cognition and self-awareness. There is also a considerable degree of convergence between genetic changes that underlie brain evolution and loci related to neuropsychiatric disorders. **(B)**. Examples of genetic alterations in the human lineage related at the same time to evolution of SCZ or ASD.

Cortical expansion, which denotes a disproportionate gain of the surface area of the cortex as compared to other regions of the brain, marks brain evolution in primates (Geschwind and Rakic, 2013; Fernández et al., 2016; Franchini, 2021). The increase in the absolute number of neurons contributes to the expansion of the cortex during evolution and is associated with restructuring of the network of connections between neuronal cells (“connectome” Figure 1A). Change in the architecture of the connectome is most likely the essential driver of the enhanced computational power of the human brain (van den Heuvel et al., 2016; Sousa et al., 2017; Ardesch et al., 2019).

Neurogenesis occurs primarily in the prenatal period of life (Villalba et al., 2021) and is ensured by activity of the neural precursors (NP), including the outer Radial Glial (oRG) cells (Rakic, 2009; Pinson and Huttner, 2021). As compared to their counterparts in the non-human primates (NHP), human oRG cells feature an enhanced capacity to self-renew. Hence, each human oRG cell can, in principle, produce more neurons than its counterpart in developing NHP brain (Florio and Huttner, 2014; Mora-Bermúdez et al., 2016; Pinson and Huttner, 2021; Pinson et al., 2022). Increase in the number of neurons is likely the cornerstone of brain evolution and is intimately linked to the reshaping of the connectome. Human connectome features larger number and diversity of areas in the cortex, and most likely evolved by enhancing the modularity, sparsity and segregation of the network (Changeux et al., 2021). The relationship between cortical expansion and changes in human connectome is, however not fully understood. According to the tethering hypothesis (Buckner and Krienen, 2013), the rapid expansion of the human cortex disrupted the organization of the connectome, and led to the establishment of new networks, which, in turn, enabled the emergence of the enhanced cognitive abilities of the human mind. Further changes in connectome were probably related to an increased intricacy of the cytoarchitecture of neurons as, for instance, in the case of pyramidal neurons (Galakhova et al., 2022). Likewise, the further specialisation and perhaps also the emergence of unique neuronal cell types (Allman et al., 2010; Banovac et al., 2021) and evolutionary changes in non-neuronal cells, including neuroglial cells, also likely contributed to changes in connectome during human brain evolution (Oberheim et al., 2009; Fields et al., 2015; Oberheim Bush and Nedergaard, 2017).

## 3 Genetic changes that link brain evolution, schizophrenia and autism spectrum disorder

Evolutionary changes in gene activity may emerge from alterations in the sequence of the *gene* (for review see Espinós et al. (2022)) or of the *DNA regulatory elements* including promoters, enhancers, silencers or insulators (for reviews see Bae et al. (2015); Silver, (2016); Liu et al. (2021)). As a result, genes which are similarly expressed across species may still differ in function due to divergence in coding sequences (Staes et al., 2017), while genes with conserved coding sequence may vary in expression level between species due to alterations in regulatory program (Prescott et al., 2015; Kanton et al., 2019; Wang et al., 2020).

According to the most recent estimates, 856 genes have undergone changes specific to the human lineage when compared to great apes (Bitar et al., 2019). Genes affected by evolution are primarily related to brain biology (71 of these loci are related to nervous system development and functions), immune system and metabolism. This most up-to-date account, is, however, most likely far from complete. Multiple gene duplications have been uncovered in the most recent human genome assembly (Nurk et al., 2022; Vollger et al., 2022). Remarkably, coding sequences of genes related to brain biology including loci implicated in nervous system development and synaptic transmission are generally conserved between humans and NHP (Dumas et al., 2021). This suggests that brain evolution might be driven primarily by differences in transcriptomes. Comparison of RNA-seq profiles of neuronal cells from the human and NHP stem cell-derived organoids and brain specimens (Kanton et al., 2019; Pollen et al., 2019; Ma et al., 2022), revealed hundreds of genes featuring altered expression in human, as compared to the NHP brain cells; these differences affected both neurons and glial cells including astrocytes, and oligodendrocytes (Kanton et al., 2019; Ma et al., 2022). While we anticipate the discovery of many more loci related to brain biology and evolution (Yamasaki et al., 2020), the already available analyses help identify several remarkable examples of human specific genes which may contribute to, and therefore bridge, human brain evolution, cognitive traits, and NDs (Figure 1).

### 3.1 Evolutionary hotspot at chromosome 1 region q21.1-2 contains genes linked with brain size, schizophrenia, and autism spectrum disorder

The cytoband q21.1-2 region at chromosome 1 is a site of a significant gene number expansion in the recent human evolution. Likewise, 1q21.1-2 appears polymorphic in the human population. Based on United Kingdom Biobank data, prevalence of copy number variations at this region is estimated at 0.027% for deletions and 0.044% for duplications (Owen et al., 2018). 1q21.1-2 encodes genes from the *NOTCH2NL* (Fiddes et al., 2018; Florio et al., 2018; Suzuki et al., 2018) and neuroblastoma breakpoint (*NBPF*) families (O’Bleness et al., 2014), both of which are genetically linked to SCZ and ASDs (Searles Quick et al., 2015; Fiddes et al., 2018; Davis et al., 2019). The 1q21.1-2 is most probably the best described example of a locus harboring genes amplified in the human lineage, that is also linked to changes in brain size and ND risk (see below).

#### 3.1.1 *NOTCH2NL* gene family

The human specific *NOTCH2NL* gene family comprises four paralogs: three protein-coding genes (*NOTCH2NL A*, *B*, and C) and a pseudogene (*NOTCH2NLR*) (Suzuki et al., 2018). *NOTCH2NL* is absent in the macaques, while gorilla and chimpanzee genomes feature only *NOTCH2NL* pseudogenes (Fiddes et al., 2018; Suzuki et al., 2018), which is most likely a product of an incomplete duplication of an essential developmental regulator *NOTCH2* (Fiddes et al., 2018). The current data suggests that, in the human lineage, the non-functional ancestral copy of *NOTCH2* was first “re-activated” to form a functional gene and subsequently multiplied producing the *NOTCH2NL* *A*, *B*, and *C* genes (Fiddes et al., 2018).

Expression of *NOTCH2NLB* favors activation of the NOTCH pathway by inhibiting cis DELTA-NOTCH interactions (Fiddes et al., 2018). Experimental data in a mouse model and in organoid system show that *NOTCH2NL* genes may have contributed to cortical expansion by enhancing the proliferative capacity of the RG cells, and by delaying the differentiation of the RG cells to neurons (Fiddes et al., 2018; Florio et al., 2018; Suzuki et al., 2018). In human adult brain, both the ancestral gene *NOTCH2* and members of *NOTCH2NL* family are expressed most strongly in astrocytes (Zhang et al., 2016; Kanton et al., 2019). Search for “NOTCH2NL” at https://brainrnaseq.org/ and https://bioinf.eva.mpg.de/shiny/sample-apps/scApeX/, Accessed September 29, 2022.

Alterations of the in the copy number of *NOTCH2NL* family genes are linked to congenital syndromes and NDs (Stone et al., 2008; Bernier et al., 2016; Macé et al., 2017). Microdeletions and microduplications of *NOTCH2NL* relate to micro- and macrocephaly respectively (Brunetti-Pierri et al., 2008; Mefford et al., 2008); likewise, loss or amplification of *NOTCH2NL* is detected in SCZ and ASD patients respectively (Pang et al., 2020).

Taken together, the emergence of the *NOTCH2NL* gene family increased the activity of NOTCH signaling pathway in the human neural stem cells which, likely by endowing them with an enhanced capacity to self-renew, contributed to the evolutionary expansion of the brain cortex in humans. Correct gene dosage of *NOTCH2NL* is essential not only to ensure appropriate brain size but also to allow proper neuronal activity (Chapman et al., 2022). Hence, the evolutionary expansion of *NOTCH2NL* gene family would result in a trade-off between an advantageous increase in one trait—a gain in brain size, and a detrimental change in another—an increased susceptibility to SCZ and ASD. It will be interesting to determine how *NOTCH2NL* gene dosage is distributed across a large human population and how the numbers (and isoforms) of *NOTCH2NL* genes correlate with brain structure and activity in ASD and SCZ.

#### 3.1.2* NBPF* gene family

The neuroblastoma breakpoint (*NBPF*) genes contain the Olduvai domain (OD), which underwent a remarkable expansion during recent human evolution (Popesco et al., 2006). Genome of the last common ancestor of humans and chimpanzees featured a total of 102 copies of ODs, chimpanzee genome contains 125 copies, while human genome contains 272 copies of ODs (O’Bleness et al., 2012). Expansion of such magnitude suggests an important function of genes containing ODs. Indeed, OD-containing proteins are known to contribute to neural progenitor proliferation (Keeney et al., 2015), and the number of OD repeats in the genome is related to severity of both ASD (Davis et al., 2014, 2015, 2019) and SCZ (Searles Quick et al., 2015). Similarly to *NOTCH2NL* genes, members of the NBFP gene family are expressed in astrocytes in humans, especially in fetal astrocytes (observation based on transcriptomic databases published in Zhang et al., (2016), Kanton et al., (2019)). Yet, the molecular function of ODs remains to be determined (Sikela and Searles Quick, 2018).

1q21.1-2 region harbors 13 genes from the *NBPF* family (O’Bleness et al., 2012). *NBPF* genes at the 1q21.1-2 region contain most (119) of the human-specific Olduvai domain repeats (Fiddes et al., 2019). *NBPF* genes lie proximally to each other at the 1q21.1-2 locus and are most likely transcriptionally co-regulated with *NOTCH2NL* genes (Fiddes et al., 2019). This genomic configuration seems to be inherently genetically unstable (Fiddes et al., 2018) and highly prone to non-allelic homologous recombination events (Chapman et al., 2022). In fact, the marked chromosomal instability of the 1q21.1-2 locus is likely caused by the presence of amplified *NOTCH2NL* and *NBPF*. This genetic configuration in turn, causes alterations in the copy number of other genes lying in q21.1-2 region including *HYDIN2 PRKAB2, FMO5, CHD1L, BCL9, ACP6, GJA5, GJA8, GPR89B* and *PDZK1*, which likely drives additional detrimental effects (Brunetti-Pierri et al., 2008; Yoon and Mao, 2021). Therefore, the local expansion of *NOTCH2NL* and *NBPF* genes may have exerted a direct effect through altering the dosage of the *NOTCH2NL* and *NBPF* genes but also an indirect impact by enhancing the probability of genetic alterations at the locus manifesting as copy number variants at the 1q21.1-2.

### 3.2 Copy number variants within the human-specific 16p11.2 locus are related to multiple NDs, including schizophrenia and autism spectrum disorder

The 600,000 bp region between breakpoints 4 and 5 (BP4-BP5) at chromosome 16 cytoband p11.2 (16p11.2) carries multiple genes, including *BOLA2*, *SLX1*, *MAZ*, and *SULT1A3*. A 95 kbp part of the region carrying *BOLA2* has undergone a human-specific duplication in the most recent human evolution, from one copy in Neanderthals and Denisovans to three to eight diploid copies in modern humans (Nuttle et al., 2016). *BOLA2* appears to be important from the evolutionary standpoint. *BOLA2* encodes a cytosolic protein implicated in the maturation of iron-sulfur proteins; it is more expressed in the human embryonic stem cells and induced pluripotent stem (iPS) cells than in their chimpanzee counterparts (Li and Outten, 2012; Banci et al., 2015). Amplification of *BOLA2* in the human lineage might protect from iron deficiency (Giannuzzi et al., 2019). A study using iPS cell models with altered copy number of the 16p11.2 region revealed that neurons obtained from iPS cells featuring 16p11.2 deletion are hyperactivated and overexpress synaptic markers compared to the isogenic control cells; neurons with 16p11.2 duplication display largely opposite characteristics (Sundberg et al., 2021).

Genes located in BP4-BP5 region of 16p11.2 are essential for proper brain activity, as evidenced by the fact that CNV in 16p11.2 are among the most frequent causes of neurodevelopmental disorders (Rein and Yan, 2020; Chung et al., 2021). Prevalence of reciprocal deletions of 16p11.2 is estimated at 1/2000, and of and reciprocal duplications at 1/1100 (Chung et al., 2021); triplications are very rare (Wallace et al., 2018).

Dosage of genes located in 16p11.2 has been linked to brain structure. Number of copies of the 16p11.2 region anticorrelates with the gray matter volume and is related to the microstructure of the regions implicated in reward, language and social cognition (Maillard et al., 2015). CNV in 16p11.2 are related to ASD, SCZ, intellectual disability, epilepsy, macrocephaly, depression, anxiety and attention deficit hyperactivity disorder (ADHD) (Rein and Yan, 2020). The nature of the mechanistic implication of the 16p11.2 locus in brain biology is, however, not clear. Despite this knowledge gap, a treatment for ASD symptoms induced by 16p11.2 deletion is currently under development. R-Baclofen has been successfully used to treat symptoms of ASD in mouse model of 16p11.2 deletion, and its clinical trials are in Phase II (Parellada et al., 2021, ClinicalTrials.gov Identifier: NCT03682978). It is likely that by determining how the evolutionary changes in the DNA sequence of 16p11.2 impacted brain biology, we will gain critical new insights into the mechanisms of NDs related to genes encoded at 16p11.2.

### 3.3 Recently evolved genes related to NDs

Comparison of the genome sequences of modern humans with Neandertals and Denisovans, the two best characterized archaic forms of humans (Green et al., 2010; Prüfer et al., 2014; Peyregne et al., 2017), allowed identification of a number of genes which underwent positive selection in humans. These include loci affected by the selective sweep, a process of genetic hitchhiking accompanied by directional selection (Green et al., 2010). Of those, *NRG3*, *CADPS2*, *AUTS2,* (Green et al., 2010) and *AMBRA1* (Prüfer et al., 2014) seem particularly interesting in the context of this review, as they have also been linked to both SCZ and ASD (Figure 1B)

**Neuregulin 3** (*NRG3*) is located in a region which has been under strong selective pressure after divergence between humans and Neanderthals (Green et al., 2010). NRG3 is an epidermal growth factor-like signaling protein acting on receptor tyrosine kinase ERBB4 (Avramopoulos, 2018). NRG3 is expressed in the developing and adult brain, in both glial and neuronal cells (Zhang et al., 2016; Karlsson et al., 2021). The NRG3-mediated signaling is important for brain development (Bartolini et al., 2017; Müller et al., 2018), and the formation of neural circuits (Exposito-Alonso et al., 2020). NRG3 was shown to promote neurite outgrowth (Rahman-Enyart et al., 2020) and control glutamate release (Wang et al., 2018). Interestingly, distinct NRG3 isoforms are expressed in the human brain at different developmental stages (Paterson et al., 2017). Importance of NRG3 for healthy brain functioning has been confirmed *in vivo* in mice with NRG3 knock-out, which display psychotic syndromes (Hayes et al., 2016). However, the mechanism by which NRG3 might affect behavior is still not fully understood.

There are 11 single nucleotide polymorphisms (SNPs) at the *NRG3* locus, of which 5 have been directly linked to risk of SCZ; 3 SNPs seem to associate with the strength of delusion symptoms in SCZ patients, but not risk of developing SCZ (Avramopoulos, 2018). The rs10748842 lies in the region affected by a human-specific selective sweep. T/T variant of this SNP is positively related to the strength of delusions in SCZ; it has also been shown to correlate with elevated expression of particular NRG3 isoform classes (II and III) in patients with mood disorders (Paterson et al., 2017). Furthermore, the genotype at rs10748842 is related to cognitive functions in SCZ patients, individuals with SCZ who carry T/T variant of this SNP display decreased cognitive deficit than patients with the T/C or C/C genotypes (Li et al., 2020; Zhou et al., 2020). According to the 1000 genomes project (www.internationalgenome.org, retrieved on 29-09-22), the T allele is the most prevalent haplotype in the human population (frequency of T = 86%, frequency of C = 14%) (Fairley et al., 2020). Such high frequency suggests that the effect of T allele is otherwise neutral or beneficial, and only exerts its effect in brains of diseased individuals.

Interestingly, SCZ patients also feature disfunctions of ERBB4 which is regulated by both NRG3 and NRG1, which has also been implicated in SCZ (Mostaid et al., 2016). EGF domains of both Nrg1 and Nrg3 promote synapse formation in mouse pyramidal neurons further highlighting the functional convergence of NRG1 and 3 (Exposito-Alonso et al., 2020). Location, and hence the activity, of NRG3 depends in its processing (Vullhorst et al., 2017). In the mouse, the unprocessed isoform accumulates in the cell body, while the processed protein, including proteolytically cleaved portions of it, are transported to other subcellular locations, the N-terminal fragment of NRG3 can for instance be transported to the cell membrane of the neurites, where it can interact with the postsynaptic ErbB4 receptors (Ahmad et al., 2022). Interestingly, the N-terminus of NRG3 is also the site of the selective sweep in modern humans as compared to Neanderthals (Green et al., 2010).

The 7q31–q33 constitutes another example of a region that underwent a positive selection in the recent human genome history. There are several protein-coding loci within the 7q31–q33 region including *RNF148*, *RNF133*, and ***CADPS*2**; the latter being particularly interesting from the clinical and evolutionary standpoints. In human, deletions as well as amplifications of the genetic interval containing *CADPS2* (Ca^2+^-dependent activator protein for secretion 2) have been associated to ASD (Bonora et al., 2014; Grabowski et al., 2017; Nagy et al., 2021). Interestingly, *CADPS2* can be aberrantly spliced in ASD in autistic patients (Sadakata et al., 2007). On the other hand, *CADPS2* is transcriptionally upregulated in SCZ patient brains (Hattori et al., 2011).

*CADPS*2, located in a region previously referred to as autism susceptibility locus 1 (*AUTS1*) (International Molecular Genetic Study of Autism Consortium, 2001). Based on the transcriptomic data from the human brain, *CADPS2* is highly expressed in mature astrocytes and neurons (Zhang et al., 2016). *CADPS2* is implicated in the regulation of secretion of synaptic and dense core vesicles (Sadakata et al., 2004). By regulating the release of vesicles, CADPS2 plays a role in the secretion of brain-derived neurotrophic factor (BDNF) and neurotrophin-3 (NT-3) thereby impacting the development and perhaps the balance between activatory and inhibitory synapses in the central nervous system (Sadakata et al., 2004, 2007; Shinoda et al., 2011). Interestingly, deletion of *CADPS*2 exerts brain region specific effects in the mouse (Shinoda et al., 2018, 2019) and affects social behavior of the animals leading to ASD-like behavior (Sadakata et al., 2007). Furthermore, the effects of *CADPS2* loss might be caused by an altered release of oxytocin in the knockout animals (Fujima et al., 2021). Altogether, gene dosage of *CADPS2* might have contributed to the evolutionary changes of brain morphology and activity. The alterations in the level of *CADPS2* might have been linked to the establishment of more intricate social structure of the human population. The defects in the proper activity *CADPS2* may alter the capacity for inter-individual interaction as observed in ASD patients.

The 5′ end of ***AUTS2*** (autism-susceptibility-gene-2) has been under strong selection after divergence between humans and Neanderthals – this portion of the *AUTS2* locus overlaps three evolutionarily accelerated non-coding regions featuring a large number (293) of SNPs differentiating modern from archaic humans (Green et al., 2010). Genetic alterations in the 5′ end of *AUTS2* locus are related to numerous neurological disorders including ASD, ADHD, micro- and macrocephaly, intellectual disability, mental retardation (Sultana et al., 2002; Kalscheuer et al., 2007; Bakkaloglu et al., 2008; Huang et al., 2010; Pinto et al., 2010; Girirajan et al., 2011; Nagamani et al., 2013) or alcohol consumption (Schumann et al., 2011). The known SNPs at the 5′ end of *AUTS2* are by and large non-coding (Oksenberg et al., 2013) and feature accelerated evolution in humans (Pollard et al., 2006b, 2006a; Prabhakar et al., 2006, see also below). It is important to stress that genetic alterations in the 3′ of *AUTS2,* with a yet unknown relevance from the evolutionary standpoint, are also related to neurological disorders and seem more penetrant than alterations in the 5’ end of the gene (Biel et al., 2022).

There are multiple animal models, including mouse and the zebrafish, amenable to study the functions of *AUST2* and this area of research experiences a rapid development with inclusion of organoid systems (Biel et al., 2022). In the mouse, *Auts2* is expressed during brain development and in multiple brain regions. AUTS2 is abundant in the cell nucleus and was shown to localize to gene promoter regions thereby suggesting its role in transcriptional regulation (Bedogni et al., 2010; Gao et al., 2014; Castanza et al., 2021). Furthermore, AUTS2 associates with RNA binding proteins in the developing mouse brain (Castanza et al., 2021). Other studies revealed the implication of AUTS2 in the regulation of cytoskeleton (Hori et al., 2014).

Experiments in mouse cell lines suggest that correct expression of *AUTS2* is essential for the regulation of neuronal differentiation (Monderer-Rothkoff et al., 2021) and functions (Hori et al., 2014). *Auts2*-deficient mice feature hyperactivation of excitatory synapses and display behavioral and cognitive changes which partially mirror symptoms observed in human autistic patients (Hori et al., 2015), providing a strong link between *AUTS2* and ASD. Transcriptomic data from acutely isolated human tissue showed strong expression of *AUTS2* in neurons as well as in astrocytes (Zhang et al., 2016; Karlsson et al., 2021), in human cerebral organoids, *AUTS2* is expressed in excitatory neurons, radial glia and intermediate progenitors (Fair et al., 2022). Heterozygous *de novo* missense mutation in *AUTS2* detected in ASD patients alters the cell cycle dynamics of neural progenitors, which could hint at the mechanism by which this gene may contribute to the regulation of brain size (Fair et al., 2022). In humans, there are two major protein isoforms of AUTS2 (Biel et al., 2022). It will be interesting to determine the molecular mechanism by which the two AUTS2 isoforms regulate gene expression in the human lineage and how they might have contributed to the evolution of the human brain.

The activating molecule in Beclin-1-regulated autophagy (***AMBRA1***) lies in a region which has undergone a selective sweep between modern and archaic humans (Prüfer et al., 2014). *AMBRA1* is an autophagy regulator (Cianfanelli et al., 2015) implicated in the development of the murine nervous system (La Barbera et al., 2019). *AMBRA1* deficiency causes neural tube malformations (Maria Fimia et al., 2007). *AMBRA1* also contributes to postnatal brain biology – neurospheres derived from *Abra* ^*+/−*^ mouse adult brain neural progenitors in the subventricular zone showed a decreased proliferation, and hence the number of neural precursor cells indicating a role of *AMBRA1* in regulation of adult neurogenesis (Yazdankhah et al., 2014). *AMBRA1* ^*+/−*^ mice feature increased apoptosis of neuronal precursors in the medial ganglionic eminence and loss of parvalbumin interneurons in the hippocampus, phenomena which correlate with a decreased inhibition/excitation ratio in female animals, most likely underlying the altered social behavior of the heterozygous animals (Nobili et al., 2018; La Barbera et al., 2019).

Based on GWAS studies, *AMBRA1* is associated with both ASD (Mitjans et al., 2017) and SCZ (Rietschel et al., 2012). The precise mechanism linking AMBRA1 to SCZ and ASD are not fully defined but most likely entail gene dosage effects as suggested by studies in the mouse model described above. An intronic SNP in *AMBRA1* (rs11819869) is related to both SCZ and impulsivity (Heinrich et al., 2013) but the functional consequences of nucleotide changes at this site are not well understood. Multiple lines of evidence suggest that gene dosage of *AMBRA1* impacts brain functions in a sexually dimorphic manner. Loss of one copy of *AMBRA1* affects primarly females and leads to ASD (Mitjans et al., 2017). SNP rs3802890 related to loss of *AMBRA1* expression in female individuals lies in an intron, which suggests it has a DNA regulatory function (Mitjans et al., 2017). Interestingly, *AMBRA1* is lost in some of microdeletions classified as cases of rare Potocki-Shaffer Syndrome, which features intellectual disability, further linking this gene to cognitive abilities (Trajkova et al., 2020).

Taken together, there is a group of genes related to ASD and SCZ that feature evolutionary changes in the coding and non-coding portions of their DNA. The sequences that link these loci to evolution are also risk loci for ASD and SCZ. Below, we outline a class of particularly interesting non-coding regulatory element variants that are linked to ASD and SCZ and may contribute to brain evolution.

## 4 Human accelerated *cis*-regulatory elements as drivers of NDs

The changes in gene regulation have long been proposed to contribute to the human-specific features of the brain (King and Wilson, 1975; Geschwind and Rakic, 2013; Reilly et al., 2015). Since genes related to brain are more conserved than other loci (Dumas et al., 2021), brain evolution might be primarily driven by transcriptome differences. Human-specific changes in regulome have been linked to corticogenesis (Reilly et al., 2015). Furthermore, regions that have undergone a selective sweep in humans after divergence from Neanderthals are enriched in enhancers (Peyregne et al., 2017). Interestingly, active chromatin regions in humans, especially those elements which are active earlier in fetal development, have been linked to cognitive ability (Yao et al., 2022).

Human-accelerated regions (HAR) are genomic segments which have undergone accelerated nucleotide sequence change in humans relative to other primates. The most recent analyses suggest that there are 3,171 HARs in the human genome (Pollard et al., 2006a, 2006b; Prabhakar et al., 2006; Bird et al., 2007; Bush and Lahn, 2008; Lindblad-Toh et al., 2011; Hubisz and Pollard, 2014; Gittelman et al., 2015) HARs overlap regulatory elements important for brain development and are particularly related to elements active in the fetal brain (Won et al., 2019; Bhattacharyya et al., 2021, 2022; Cheung et al., 2022). Recently, massively parallel assay for regulatory element activity showed that 49% of HARs feature neurodevelopmental enhancer activity in human neural cells *in vitro* (Girskis et al., 2021).

Chromatin conformation capture experiments help to link putative enhancers with their cognate promoters and can help to predict the functions of enhancers. HARs are not only preferentially located near genes which are dosage-sensitive, sequence-constrained, and associated with ASD and SCZ (Xu et al., 2015; Doan et al., 2016), but also physically interact with promoters of genes implicated in brain development (Won et al., 2019). Prominent examples of loci likely regulated by HARs and implicated in ASD and SCZ include *AUTS2*, *CDKL5*, *FOXP1*, *MEF2C*, *NRXN1*, and *SMARCA2* (Doan et al., 2016).

A more detailed analysis of the evolutionarily changed HARs can improve our understanding of the interplay between evolution and NDs. Three intronic regions between exons 1 and 4 at the *AUTS2* locus: HAR31, HACNS174, and HACNS369 have been found to be significantly accelerated in humans when compared to primates (Pollard et al., 2006a, 2006b; Prabhakar et al., 2006). HACNS369 is of particular interest as it lies within a region that features signs of human-Neandertal sweep and at the same time alteration in ASD patients (Oksenberg et al., 2013). Transgene reporter assays indicate that HACNS369 can act as an enhancer in the developing midbrain of zebrafish (Oksenberg et al., 2013). Altogether, this data suggest that human specific regulatory element at the *AUTS2* locus may contribute to brain evolution and susceptibility to ASD.

Some studies highlight that HARs seem to relate more prominently to genes previously implicated in SCZ than with other NDs such as ASD (Cheung et al., 2022). However, the meaning and relevance of this observation seems unclear. While genome-wide studies discussed above present interesting observations on a link between evolution of human regulome and SCZ and ADSs, it is also important to stress that the species-specific, recently evolved enhancers are variable between individuals and tend to have minor impact on gene expression (Castelijns et al., 2020). Altogether these observations reinforce the need for extensive functional validation of the human specific and disease associated regulatory variants in the human genome.

## 5 Summary—Perspectives and future avenues for research

One of the essential questions in neurobiology is how the genome changed to allow the increased intellectual abilities of humans compared to NHP. The discovery of the human-specific regulome of the nervous system is likely to provide important piece of information to solve this question (Yao et al., 2022). Addressing it will also likely help us understand the genetic bases of diseases that affect human specific features of mind. Above, we outlined several genes that are related to human brain evolution and which at the same time are liked to ASD and SCZ. These loci affect neurogenesis (*NOTCHNLA2, AUTS2, AMBRA1)*, brain connectivity and synaptic transmission *(NRG3, CADPS2)*, it will be fascinating to decipher the transcriptional regulatory networks that orchestrate the expression of these genes in various neural cells and understand the contribution of these loci to the development and the functions of the central nervous system in humans.

There are multiple newly discovered coding and non-coding regions implicated in SCZ, ASD and other ND that await further analysis from the evolutionary and functional standpoints (Demontis et al., 2019; Grove et al., 2019; Howard et al., 2019; Mullins et al., 2021; Wightman et al., 2021; Trubetskoy et al., 2022). However, to achieve an in-depth understanding of the contribution of non-coding sequences to NDs (and any other diseases for that matter) it will be essential to improve assemblies and annotations of the NHP genomes (Nurk et al., 2022; Vollger et al., 2022). Likewise, it will be beneficial to include a larger cohort of human and NHP genomes in the analysis (Castelijns et al., 2020), and improve protocols of *in-vitro* and *in-vivo* systems to assess the contribution of genetic variants in distinct cell types present in the human brain.
